# Religious practices and long-term survival after hospital discharge for an acute coronary syndrome

**DOI:** 10.1371/journal.pone.0223442

**Published:** 2019-10-04

**Authors:** Hawa O. Abu, Kate L. Lapane, Molly E. Waring, Christine M. Ulbricht, Randolph S. Devereaux, David D. McManus, Jeroan J. Allison, Catarina I. Kiefe, Robert J. Goldberg

**Affiliations:** 1 Department of Population and Quantitative Health Sciences, University of Massachusetts Medical School, Worcester, Massachusetts, United States of America; 2 Department of Allied Health Sciences, University of Connecticut, Storrs, Connecticut, United States of America; 3 Department of Community Medicine, Mercer University School of Medicine, Macon, Georgia, United States of America; 4 Division of Cardiovascular Medicine, Department of Medicine, University of Massachusetts Medical School, Worcester, Massachusetts, United States of America; Linkoping University, SWEDEN

## Abstract

**Background:**

Prior studies of healthy populations have found religious practices to be associated with survival. However, no contemporary studies have examined whether religiosity influences survival among patients discharged from the hospital after an acute coronary syndrome (ACS). The present study examined the relationship between religious practices and 2-year all-cause mortality among hospital survivors of an ACS.

**Methods:**

Patients hospitalized for an ACS were recruited from 6 medical centers in Massachusetts and Georgia between 2011 and 2013. Study participants self-reported three items assessing religiosity: strength/comfort from religion, petition prayers for health, and awareness of intercessory prayers by others. All cause-mortality within 2-years of hospital discharge was ascertained by review of medical records at participating study hospitals and from death certificates. Cox proportional hazards models were used to estimate the multivariable adjusted risk of 2-year all-cause mortality.

**Results:**

Participants (n = 2,068) were on average 61 years old, 34% were women, and 81% were non-Hispanic White. Approximately 85% derived strength/comfort from religion, 61% prayed for their health, and 89% were aware of intercessions. Overall, 6% died within 2 years post-discharge. After adjusting for sociodemographic variables (age, sex, and race/ethnicity), petition prayers were associated with an increased risk of 2-year all-cause mortality (HR: 1.64; 95% CI: 1.01–2.66). With further adjustment for several clinical and psychosocial measures, this association was no longer statistically significant. Strength and comfort from religion and intercessory prayers were not significantly associated with mortality.

**Conclusions:**

Most ACS survivors acknowledge deriving strength and comfort from religion, praying for their health, and intercessions made by others for their health. Although the reported religious practices were not associated with post-discharge survival after multivariable adjustment, acknowledging that patients utilize their religious beliefs and practices as strategies to improve their health would ensure a more holistic approach to patient management and promote cultural competence in healthcare.

## 1. Introduction

The acute coronary syndromes (ACS) are a life-threatening condition which account for approximately one-half of all cardiovascular disease related mortality and upwards of one-third of all deaths globally [1,2]. Although the role of sociodemographic, psychosocial and clinical variables have been extensively studied in relation to all-cause mortality after an ACS [3–5], little is known about whether patients’ religious beliefs and practices influence overall survival after hospital discharge for an ACS.

Religiosity influences both cardiovascular and non-cardiovascular health outcomes through a variety of psychological, behavioral, and social pathways [6]. Patients may find considerable meaning and purpose in religion after stressful life events, which could facilitate coping and adaptation with illness which may be accompanied by higher levels of well-being and optimism [6, 7]. These psychological processes have been shown to favorably affect patient’s inflammatory, endocrine, and autonomic functions [6,8,9]. Moreover, some religious doctrines encourage positive self-care practices such as refraining from unhealthy lifestyle behaviors which promote overall health status [6,7]. Religiosity may also favorably affect overall wellbeing through social pathways by promoting involvement in communities of faith and nurturing positive social attitudes and support networks, which may reinforce emotional well-being [6, 10]. Despite these benefits of religiosity on health, certain religious beliefs and practices may contradict recommendations by medical professionals and interfere with patient engagement with their healthcare [11] buttressing the need for better understanding of how religiosity may influence patient health outcomes and survival.

Although religiosity has been associated with survival in population-based studies of healthy individuals [12,13], inconclusive findings about the influence of religiosity on overall survival among patients with cardiovascular disease persist. Published investigations examining the association between religious involvement and health outcomes among patients with underlying cardiovascular disease have been cross sectional in nature, used small sample sizes, lacked adjustment for potentially confounding prognostic variables, or used a single item to assess religiosity, which may not adequately reflect the full extent of an individual’s religiosity [14–16]. The objective of the present study is to examine the association between religiosity and 2-year all-cause mortality after hospital discharge for an ACS using data from a large sociodemographically diverse cohort of ACS survivors. We hypothesized that patients who reported religious engagement would have lower death rates in the 2-year period after hospital discharge for an ACS compared to patients without reports of religious engagement. For purposes of the present study, religiosity is defined in terms of religious beliefs and practices including the use of personal or intercessory prayers for health and deriving strength and comfort from religion [17,18].

## 2. Methods

### 2.1. Study design and ethics approval

The Transitions, Risks and Actions in Coronary Events Center for Outcomes Research and Education (TRACE-CORE) Study used a multi-center prospective cohort design to recruit 2,174 patients hospitalized with an ACS at 6 medical centers in Central Massachusetts and Georgia between April 2011 and May 2013 [19,20]. Eligibility for study enrolment included adults aged ≥21 years who had a confirmed ACS and were discharged alive after the index acute hospitalization. The Institutional Review Boards at the study participating sites (University and Memorial that comprise the University of Massachusetts Memorial Medical Center, St. Vincent Hospital, Northside and Piedmont Hospitals, Community hospitals in Atlanta affiliated with Kaiser Permanente Georgia, and the Medical Center of Central Georgia) approved this investigation. Written informed consent was obtained from each participant before enrolment into the study.

Trained study staff abstracted data from hospital medical records and conducted a computer assisted in-person interview during the index hospitalization or by telephone within 72 hours of discharge. Additional follow up telephone interviews were conducted at 1, 3, 6, and 12 months following hospital discharge.

In the present study, we included only validated cases of an ACS (n = 2,122) categorized as either unstable angina, non-ST segment elevation myocardial infarction (NSTEMI), or ST-segment elevation myocardial infarction (STEMI) based on standard criteria [21]. Patients without information on the three items assessing religiosity (n = 54) were excluded, resulting in an analytic sample of 2,068 patients with a confirmed ACS.

### 2.2. Measures of religiosity

During the index hospitalization for an ACS, patients self-reported their religious experience based on 3 items assessing religiosity. A question adapted from the Fetzer Institute's Brief Multidimensional Measure of Religiousness/Spirituality [17], asked: “How much is religion a source of strength and comfort to you?”. Response options included “a great deal”, “a little”, “some”, and “none” (referent group). Responses of “some” or “little” were combined for analysis as few events of readmission and mortality occurred in these categories. The second item assessed petition prayers for health by asking, “Do you use prayer specifically for your health?” with response options “Yes” or “No” (referent). The third item on intercessory prayers for health asked: “Do you know of others outside of your family who are praying for your health?”, with responses “Yes” or “No” (referent). The items assessing petition and intercessory prayers for health were derived from the 2002 National Health Interview Survey (NHIS) [18].

### 2.3. Assessment of mortality

The primary study outcome was death from any cause within 2 years after hospital discharge for an ACS. All cause-mortality within 2-years of hospital discharge was ascertained by the review of medical records at participating study hospitals and death certificates at local, state, and national vital registry records. In our analysis, we examined all-cause mortality as our principal study endpoint because we sought to examine whether, among patients with cardiovascular disease, their religious beliefs and practices may influence their overall survival.

### 2.4. Participant baseline characteristics

We collected information on patient’s sociodemographic variables including age, sex, race/ethnicity (non-Hispanic White, non-Hispanic Black or Hispanic), level of education, health literacy and marital and employment status. Participants were asked how confident they were in filling out health forms; they were considered to have low health literacy if they reported having little or no confidence in completing these forms [22].

Psychosocial measures included quality of life, perceived stress, symptoms of anxiety and depression, patient activation, and cognitive impairment. Health Related Quality of Life (HRQOL) was assessed using the SF-36®v2 Health Survey with norm-based physical and mental well-being component summary scores ranging from 0–100 [23]. Disease-specific HRQOL was measured with the Seattle Angina Questionnaire (SAQ) quality of life subscale with scores ranging from 0–100 [24]. Higher scores from the SF-36 and SAQ indicate better HRQOL [23, 24] The 4-item Perceived Stress Scale (PSS4) assessed the extent to which patients found their lives “uncontrollable, unpredictable and overloading” in the preceding month [25]. The 7-item Generalized Anxiety Disorder questionnaire (GAD) measured symptoms of anxiety with scores of 5–9, 10–14, and >14 corresponding to mild, moderate, and severe anxiety, respectively [26]. Symptoms of depression were assessed with the 9-item Patient Health Questionnaire (PHQ-9) with scores of 5–9, 10–14, 15–19, and ≥20 corresponding with mild, moderate, moderately severe, and severe depression, respectively [27]. The extent of patient’s knowledge, confidence, and skills in managing their disease was assessed with the 6-item patient Activation Measure (PAM-6) [28]. Cognitive status at 1 month after hospital discharge was examined using the 11-item Telephone Interview for Cognitive Status (TICS), with scores ranging from 0–41, and categorized as ≥ 33, 26–32, and ≤25, indicating intact cognition, ambiguous, and moderate to severe cognitive impairment respectively [29].

Behavioral variables included patient’s dietary reports, physical activity measures, and a history of cigarette smoking and alcohol use. We used a series of questions from the Women’s Health initiative to assess participants’ frequency, intensity, and duration of physical activity [30]. Diet was briefly assessed with an eight-item food frequency questionnaire “Starting the Conversation” designed for implementation in health-promotion and primary care settings [31]. From the eight items, a summary score (range 0–16) was derived with lower scores reflecting a healthy diet and higher scores indicating need for dietary improvement. [31]. Clinical characteristics included ACS subtype (STEMI, NSTEMI, unstable angina), previously diagnosed cardiovascular and non-cardiovascular co-morbidities (e.g. heart failure, hypertension, stroke, arthritis, anemia, cancer, chronic lung disease, and chronic kidney disease), in-hospital procedures (coronary artery by-pass surgery, percutaneous coronary intervention), clinical complications (e.g., atrial fibrillation, ventricular fibrillation/tachycardia, bleeding), receipt of cardiac and non-cardiac medications during hospitalization, length of hospital stay, and referral for cardiac rehabilitation. The Global Registry of Acute Coronary Events (GRACE) mortality risk score (2.0) was calculated using data on patient’s age, heart rate, systolic blood pressure, elevated cardiac enzymes, ST-segment changes, serum creatinine levels, and the presence of heart failure and cardiac arrest at the time of hospital admission [32]. Higher GRACE risk scores are predictive of a greater risk of mortality [32].

### 2.5. Statistical analysis

Descriptive statistics were used to compare patients’ baseline characteristics in relation to their degree of religiosity. We used unpaired t-tests and ANOVA for between group comparisons for continuous variables, and Chi-square and Kruskal Wallis tests to compare differences across categorical variables. Kaplan-Meier survival curves were constructed to examine the unadjusted relationship between each measure of religiosity and 2-year survival after hospital discharge for an ACS. The log-rank test was used to determine the statistical significance of between group differences in post-discharge survival across the 3 measures of religiosity. Cox-proportional hazards regression models estimated the unadjusted and multivariable adjusted hazard ratios (HR) with accompanying 95% confidence intervals (CIs). We inspected Schoenfeld residuals and log-log plots to ascertain that the measures of religiosity and baseline covariates satisfied the proportional hazards assumption. The proportional hazards assumption was sufficiently met by the exposure variables and covariates included in the regression models.

For multivariable adjustment we included all 3 measures of religiosity in the Cox regression models. Multicollinearity was tested and ruled out with a variance inflation factor (VIF) of ≥3 to determine the presence of correlation between the measures of religiosity. We observed no collinearity (VIF = 2.29) which enhanced the development of an all-inclusive model. The choice of various confounding variables to be included in the models was based on clinical judgement and factors known to be associated with religiosity. Other potential confounders were tested to determine if their presence in the model changed the religiosity effect estimates by more than 10% [33]. Potential mediators such as participant lifestyle behaviors were not adjusted for in the regression models. To understand the impact of adjustment on effect estimates by different groups of variables, sociodemographic characteristics (age, sex, and race/ethnicity) were first adjusted for in the models. Next, clinical measures (length of index hospitalization, type of ACS, receipt of reperfusion therapy, and GRACE-risk score) were included in the models. Finally, psychosocial measures including symptoms of depression, anxiety, and the physical component of the SF-36 QOL measure were adjusted for in our logistic regression models in which 2-year mortality was the principal study outcome.

We conducted a stratified analysis by geographic location of the study sites (Central Massachusetts and Georgia) since religiosity may differ across these regions and varying clinical practices across our participating sites may influence mortality after hospital discharge for an ACS.

## 3. Results

### 3.1. Participant baseline characteristics

The average age of study participants was 61 years, 34% were women, and 81% were non-Hispanic White. Overall, 55% percent of patients were diagnosed with an NSTEMI, 30% with unstable angina, and 15% with a STEMI. Approximately 80% of patients had ≥1 previously diagnosed comorbidity, 52% were hospitalized for more than 3 days, and two-thirds of participants had undergone a percutaneous coronary intervention (PCI) during their index hospitalization for an ACS.

### 3.2. Participant characteristics according to measures of religiosity

Approximately one-half of the study population (52%) reported obtaining a great deal of strength and comfort from religion, 34% reported obtaining some or little comfort, and 14% indicated none. Approximately 60% of patients prayed for their health and 89% were aware of others praying for their health. Participants who endorsed each measure of religiosity were more likely to be women and non-Hispanic Blacks, reported higher levels of perceived stress and had lower QOL scores compared with their respective counterparts who did not respond in the affirmative to each religiosity measure. A higher prevalence of severe symptoms of depression and anxiety was observed among those who made petition prayers for their health (versus those who did not pray for their health) and those aware of intercessions made for their health by others (as compared with those unaware of intercessions made for their health). Patients who had others praying for their health had greater social support than those without others praying for their health (Table 1).

**Table 1:** Baseline sociodemographic and psychosocial characteristics of hospital survivors of an acute coronary syndrome by religiosity measures.

Characteristics	Strength and comfort from Religion				Petition prayers for health		Intercessory prayers for health	
	A great deal (n = 1,084)	Little/Some (n = 682)	None (n = 302)	Yes (n = 1,259)	No (n = 809)	Yes (n = 1,837)	No (n = 231)
Age (mean, years (sd))	62.7 (11.2)	59.7 (11.0)	59.4 (11.6)*	62.2 (11.2)	59.7 (11.2)*	61.1 (11.3)	62.2 (11.0)
Age (years, %)							
<55	25.5	33.9	35.2	27.3	33.5	30.3	24.7
55–64	29.9	33.4	32.2	30.3	33.0	31.3	32.5
≥ 65	44.6	32.7	32.6	42.4	33.5	38.4	42.8
Women (%)***	44.4	24.5	16.5	41.3	21.9	35.0	24.0
Married (%)	54.8	62.9	60.1*	56.3	62.0*	59.2	53.0
Race/Ethnicity (%)***							
Non-Hispanic Whites	72.2	88.8	95.7	74.1	92.0	80.2	88.0
Non-Hispanic Blacks	24.1	8.5	2.3	22.3	5.7	16.6	9.4
Hispanics	3.7	2.7	2.0	3.6	2.3	3.2	2.6
Education (≤ high school)	47.9	45.6	46.5	49.5	43.0*	46.5	50.7
Low health literacy (%)	38.0	34.4	32.4	35.8	38.8	37.1	34.3
Unemployed/retired (%)	65.0	52.4	51.2*	64.2	50.6*	58.7	59.7
Uninsured (%)	8.5	10.3	12.0	9.6	9.5	9.8	8.2
High perceived stress (%) ^†^ ***	50.6	49.3	38.1*	53.6	40.2*	49.4	39.8*
Depressive Symptoms (%) ^§^							
None	48.6	51.1	57.3	46.1	58.0*	49.6	59.1*
Mild	27.6	27.0	24.1	27.5	26.0	27.6	21.8
Moderate	13.0	13.4	12.9	14.9	10.2	13.1	13.5
Moderately Severe/Severe	10.8	8.5	5.7	11.5	5.8	9.7	5.6
Anxiety Symptoms (%) ^‡^							
None	49.1	51.2	56.0	46.5	57.4*	49.8	58.8*
Mild	21.4	22.5	22.5	22.0	21.8	22.5	16.7
Moderate/Severe	29.5	26.3	21.5	31.5	20.8	26.7	24.5
Low social support (%)	4.7	4.4	7.7	4.8	5.3	4.3	10.9*
Cognitive impairment (%) ^¶^	26.3	16.3	8.3*	26.0	11.6*	20.9	16.5
Patient Activation, Mean (SD)	60.0 (15.2)	60.0 (15.7)	58.2 (14.2)	59.6 (15.4)	59.9 (15.0)	60.1 (15.2)	57.3 (15.1)*
SF-36®v2 PCS, median (IQR) ***	40.0(30.6, 48.1)	43.8(35.5, 50.8)	44.7(37.1, 51.7)	40.6(31.0, 48.8)	43.9(36.3, 50.7)	41.6(32.6, 49.5)	45.6(37.0, 52.0)
SF-36®v2 MCS, median (IQR)***	49.9(37.5, 57.5)	50.7(39.5, 56.8)	52.1(42.9, 57.2)	48.9(36.8, 56.6)	52.1(42.9, 57.7)	50.3(38.8, 57.1)	52.1(42.5, 57.7)
SAQ QOL score, median (IQR)***	58.3(41.7, 83.3)	58.3(41.7, 83.3)	66.7(41.7, 83.3)	58.3(41.7, 83.3)	66.7(41.7, 83.3)	58.3(41.7, 83.3)	66.7(41.7, 83.3)

Abbreviations: PCS, Physical Component Summary; MCS, Mental Component Summary; SAQ QOL, Seattle Angina Questionnaire Quality of Life

* P<0.05 across response categories for respective religiosity measure

*** P<0.05 across response categories for all 3 religiosity measures

† Cohen’s Perceived Stress Scale Score (≥4 median, high perceived stress)

§ PHQ-9 Patient Health Questionnaire 9 item score (5–9 mild; 10–14 moderate; 15–19 moderately severe; and ≥20 severe depression)

‡ GAD-7 General Anxiety Disorder 7 item score (5–9 mild; 10–14 moderate; ≥15 severe anxiety)

^¶^TICS Telephone Interview for Cognitive Status Score (≤ 28 impaired)

For each measure of religiosity, a greater proportion of participants who provided affirmative responses were non-smokers and non-users of alcohol and were less likely to have been referred to cardiac rehabilitation than patients who did not acknowledge any of the religiosity measures (Table 2). Patients who derived strength and comfort from religion and prayed for their health were on average older, had a higher prevalence of previously diagnosed comorbidities, moderate to severe cognitive impairment, higher GRACE risk scores, and were more likely to have undergone CABG surgery during their index hospitalization compared with those who did not derive strength and comfort from religion or who prayed for their health (p<0.05 for all comparisons).

**Table 2:** Baseline behavioral and clinical characteristics of hospital survivors of an acute coronary syndrome according to measures of religiosity.

Characteristics	Strength and comfort from Religion				Petition prayers for health		Intercessory prayers for health	
	A great deal (n = 1,084)	Little/Some (n = 682)	None (n = 302)	Yes (n = 1,259)	No (n = 809)	Yes (n = 1,837)	No (n = 231)
Alcohol use (%) ***							
No alcohol use	52.6	33.3	33.3	50.4	32.7	44.1	38.3
Rare/occasional	31.4	40.0	37.7	31.9	40.2	35.3	33.5
Moderate/heavy	16.0	26.7	29.0	17.7	27.1	20.6	28.2
Smoking status (%) ***							
Non-smoker	34.0	27.4	26.6	34.2	25.5	31.9	21.7
Prior smoker	45.7	47.4	41.9	44.7	47.2	46.1	42.4
Current smoker	20.3	25.2	31.5	21.1	27.3	22.0	35.9
Physical Activity							
Total MET score (hr/week, Mean (SD))	3.5 (6.1)	4.8 (8.2)	5.1 (8.3) *	3.6 (6.4)	5.0 (8.3) *	4.1 (7.1)	4.6 (7.6)
Diet—STC summary score (Mean (SD))	6.7 (2.6)	6.7 (2.6)	6.7 (2.6)	6.6 (2.7)	6.8 (2.6)	6.7 (2.7)	6.5 (2.5)
Physiologic measures at admission							
Heart rate (beat/min, median, IQR)	75 (65–88)	75(65–88)	75 (64–86)	76 (65–88)	74 (64–87)	75 (65–88)	76 (64–87)
Systolic BP (mmHg, median, IQR)	140 (123–156)	141 (125–157)	141 (126–158)	140 (124–156)	142 (125–158)	140 (124–157)	140 (125–153)
Diastolic BP (mmHg, median, IQR)	78 (67–88)	80 (71–91)	81 (71–93) *	78 (68–89)	80(70–91) *	79 (69–90)	79 (68–91)
Laboratory values at admission							
Creatinine (mg/dl, median, IQR)	1.0 (0.8–1.2)	1.0 (0.8–1.2)	1.0 (0.8–1.1)	1.0 (0.8–1.2)	1.0 (0.8–1.2)	1.0 (0.8–1.2)	1.0 (0.8–1.2)
Glucose (mg/dl, median, IQR)	126 (104–168)	127 (106–168)	127 (106–180)	125 (104–168)	128 (107–171)	127 (105–168)	124 (107–170)
Potassium (mmol/l, median, IQR) ***	4.0 (3.7–4.3)	4.1 (3.7–4.4)	4.1 (3.8–4.4)	4.0 (3.7–4.3)	4.1 (3.8–4.4)	4.0 (3.7–4.3)	4.2 (3.8–4.4)
WBC count (10^9^cell/L, median, IQR)	8.1 (6.6–10.4)	8.9 (7.0–11.2)	9.0 (7.3–11.6) *	8.2 (6.6–10.7)	8.9 (7.1–11.2) *	8.5 (6.8–10.8)	8.7 (6.9–11.4)
GRACE risk score, mean (SD)^#^	99.4	92.4	90.1*	97.7	91.8*	95.0	97.4
Co-morbidities at admission (%)							
Anemia	4.6	1.8	3.3	5.2	4.1	4.7	5.2
Arthritis	11.7	17.3	16.2*	22.4	15.8*	19.3	23.8
Cancer	13.6	10.6	8.9*	13.2	10.0*	12.0	11.7
Chronic lung disease	19.2	17.0	15.9	18.7	16.8	18.3	15.1
Chronic kidney disease	12.8	9.2	7.3*	11.9	9.2*	10.7	11.7
Congestive heart failure	17.0	12.8	9.0*	16.6	11.0*	14.2	16.5
Diabetes mellitus	34.6	28.3	26.9*	33.4	28.4*	32.0	27.3
Hypertension	81.0	70.8	69.1*	79.5	70.4*	76.3	73.2
Stroke	7.2	4.4	1.7*	6.8	3.3*	5.7	3.9
Type of ACS (%)							
Unstable Angina	32.1	27.6	28.6*	31.7	27.6*	30.4	27.3
NSTEMI	55.9	56.2	49.5	56.4	53.0	54.8	57.1
STEMI	12.0	16.2	21.9	11.9	19.4	14.8	15.6
Reperfusion therapy (%)							
Medical treatment	23.7	19.4	17.3*	23.1	18.6*	21.5	20.3
PCI	61.5	68.3	72.7	62.7	69.7	64.6	71.9
CABG	14.8	12.3	10.0	14.2	11.7	13.9	7.8
In-hospital medications (%)							
ACEI/ARB	60.1	62.9	64.2	61.7	61.4	60.5	70.1*
Anticoagulants	69.6	75.4	78.5*	71.0	75.6*	72.3	76.6
Antidepressants	21.9	18.6	14.9	21.7	16.8*	19.9	18.6
Antidiabetic agents	12.9	9.8	9.3	12.6	9.4*	11.3	11.7
Aspirin	96.1	97.5	98.3	96.3	97.8	96.6	99.6*
Beta-Blockers	89.8	90.8	93.0	89.7	92.0	90.3	93.1
Lipid lowering medication	87.5	93.4	90.1*	89.3	90.7	89.8	90.0
NSAIDS	12.0	11.4	11.6	11.2	12.1	11.9	10.4
P2Y_12_ receptor antagonists	80.4	82.5	88.4*	80.4	85.3*	81.9	85.3
In-hospital complications (%)							
Acute kidney injury	8.7	3.2	1.0*	7.6	2.8*	6.0	3.5
Atrial fibrillation	8.7	5.7	7.0	8.3	6.1*	7.3	8.2
Bleeding	1.3	1.8	1.0	1.3	1.6	1.5	0.4
Ventricular tachycardia/fibrillation	4.8	4.4	5.6	4.8	4.8	4.8	4.3
Length of hospital stay, ≥4 days (%)***	56.8	49.6	43.9	56.3	46.7	53.7	43.3
Cardiac rehabilitation referral (%)***	28.7	44.6	55.5	30.9	48.9	36.4	50.4

Abbreviations: BP, blood pressure; WBC, white blood cell; NSTEMI, Non-ST segment elevation myocardial infarction; STEMI, ST segment elevation myocardial infarction; PCI, percutaneous coronary intervention; CABG, coronary artery by-pass graft; MET, Metabolic Equivalent derived from total energy expenditure from walking and vigorous exercise weekly; STC, Start the Conversation, Eight item dietary measure

* P<0.05 across response categories for respective religiosity measure

*** P<0.05 across response categories for all 3 religiosity measures

# GRACE risk score estimates mortality risk at 1 and 3 years after ACS admission. Score ranges from 0 to 263, higher scores are worse. Derived from data on age, heart rate, systolic blood pressure, ST segment changes, cardiac biomarkers, serum creatinine or history of renal dysfunction, Killip class or diuretic use, cardiac arrest during hospitalization for ACS.

### 3.3. Association between religiosity and 2-year all-cause mortality

**Fig 1:**
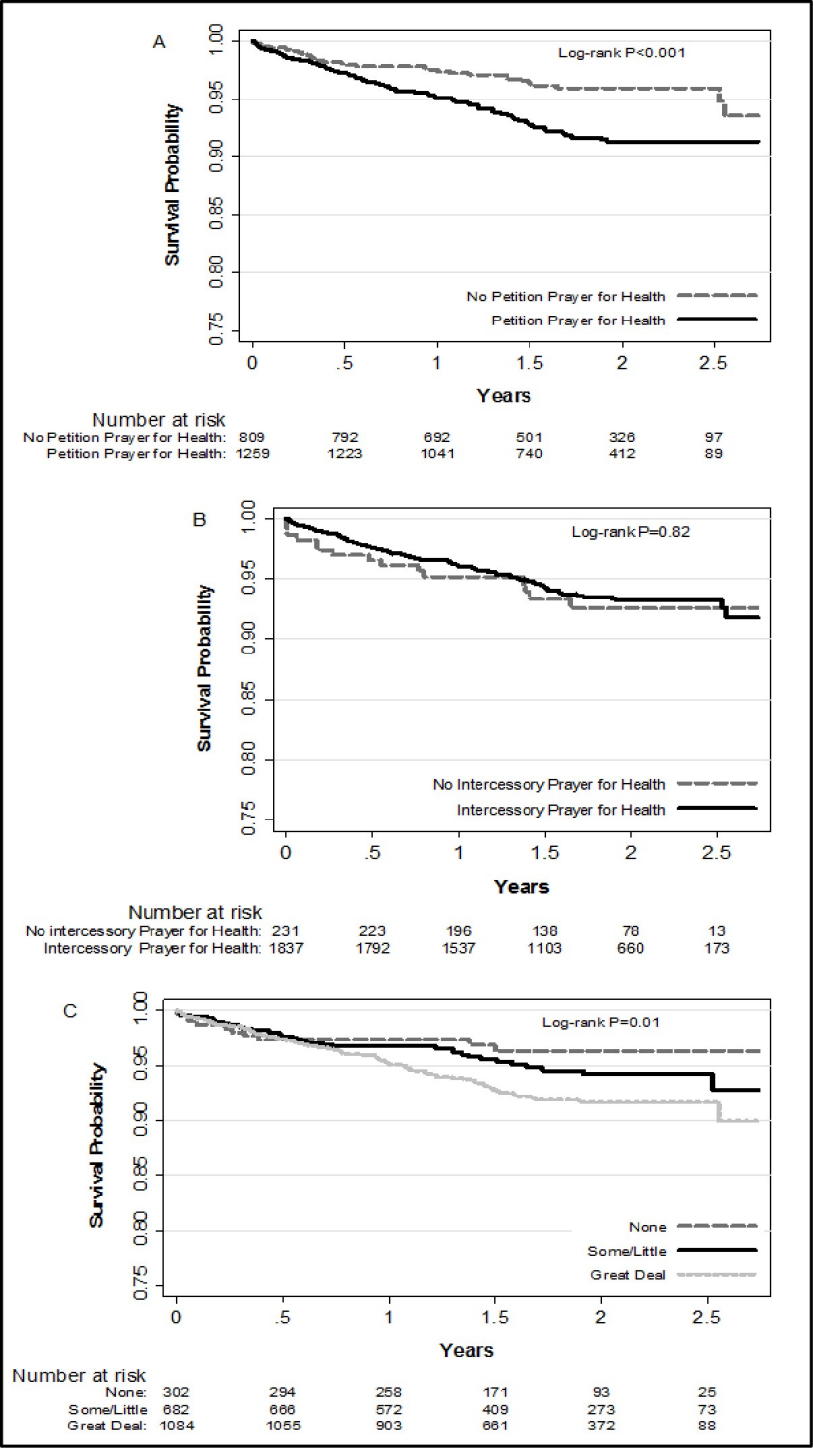
Kaplan-Meier survival curves for patients who survive at least 2 years after hospital discharge for an acute coronary syndrome according to the three measures of religiosity. (A) No petition prayer for health/Petition prayer for health. (B) No intercessory prayer for health/Intercessory prayer for health. (C) Strength and comfort from religion (None, Some/Little, Great Deal).

Of the 2,068 study participants, a total of 123 deaths (5.9%) occurred within 2 years of hospital discharge for an ACS. Patients who derived a great of strength and comfort from religion had the highest unadjusted risk of dying within 2 years compared with those who derived little/some and none, respectively (7.3% vs. 5.0% vs 3.3%, p = 0.01). Patients who prayed for their health experienced a higher unadjusted risk of dying within 2 years compared with those who did not offer petition prayers for their health (8.9% vs. 4.4%, p = 0.001). Participants who were aware of intercessions made for their health experienced a non-significantly lower crude risk of dying over our 2-year follow-up period (5.9% vs. 6.5%, p = 0.71) (Fig 1). We did not observe any significant associations between a great deal of strength and comfort from religion (hazard ratio [HR]: 1.25; 95% CI: 0.57–2.73) nor intercessory prayers for health (HR:0.80; 95% CI: 0.43–1.48) with all-cause 2-year mortality in the multivariable adjusted regression models. After accounting for sociodemographic characteristics (age, sex, and race/ethnicity), petition prayer for health was associated with an increased risk of 2-year all-cause mortality (HR: 1.64; 95% CI: 1.01–2.66). With further adjustment for several clinical and psychosocial variables, however, the association between petition prayers and 2-year all-cause mortality was no longer statistically significant (HR: 1.24; 95% CI: 0.75–2.02; Table 3).

**Table 3:** Risk of 2-year all-cause mortality among patients discharged from the hospital following an acute coronary syndrome according to measures of religiosity.

Religiosity measures	Deaths (n)	Unadjusted Model HR (95% CI)	Model 1* HR (95% CI)	Model 2** HR (95% CI)	Model 3*** HR (95% CI)
Strength and Comfort from Religion					
A great deal	79	1.64 (0.76–3.52)	1.39 (0.64–3.00)	1.07 (0.50–2.28)	1.25 (0.57–2.73)
A little/Some	34	1.32 (0.63–2.78)	1.31 (0.62–2.75)	1.03 (0.48–2.19)	1.26 (0.57–2.74)
None	10	Referent	Referent	Referent	Referent
Petition prayers for health					
Yes	92	1.81 (1.11–2.95)	1.64 (1.01–2.66)	1.50 (0.93–2.42)	1.24 (0.75–2.02)
No	31	Referent	Referent	Referent	Referent
Intercessory prayers for health					
Yes	108	0.60 (0.33–1.07)	0.69 (0.38–1.23)	0.75 (0.41–1.35)	0.80 (0.43–1.48)
No	15	Referent	Referent	Referent	Referent

Note: The unadjusted model includes all three measures of religiosity

*Model 1: Adjusted for sociodemographic variables: age, sex, and race/ethnicity

**Model 2: Adjusted for the sociodemographic variables in Model 1, and clinical variables: type of acute coronary syndrome, receipt of reperfusion therapy, length of index hospitalization, comorbidities at admission, GRACE risk score

***Model 3: Adjusted for the sociodemographic and clinic variables in Model 2, and psychosocial variables: symptoms of depression and physical component of SF-36 Quality of Life measure

In examining the association between religiosity and post-discharge mortality stratified by the two study sites, there was minimal evidence of heterogeneity in the site-specific results.

## 4. Discussion

In this prospective investigation of over 2,000 hospital survivors of an ACS, more than one-half reported receipt of strength and comfort from religion, prayed for their health, and were aware of intercessory prayers made for their health. Overall, 6% of study participants died within 2 years of hospital discharge. Patients who indicated religious engagement were more likely to be non-smokers and report moderate to no use of alcohol. A majority of participants who reported using prayers for their health had greater social support, but they tended to have more severe symptoms of depression and anxiety compared with those who did not use prayers for their health. After multivariable adjustment for several potentially confounding sociodemographic, clinical, and psychosocial characteristics, contrary to our hypothesis, none of the reports of religiosity were significantly associated with lower 2-year all-cause mortality.

### 4.1. Extent of religious practices for health

The high prevalence of religious engagement reported by our study participants is consistent with an earlier investigation of 151 US adults who underwent CABG surgery; 68% of these individuals reported that they used private prayer to cope following CABG surgery [34]. Similarly, a nationally representative study using data from the National Health Interview Survey (NHIS) found that 69% of 2,262 cancer survivors prayed for their health during recovery [35]. In addition, a population-based report from the Pew Research Center in 2014, which surveyed more than 35,000 adults in the US about their religious beliefs and practices showed that more than one-half of Americans acknowledged that religion was very important in their lives and prayed daily [36] Our study findings and results from prior research reinforce that many patients utilize religious beliefs and practices as part of their strategies for managing their health. Hence, there remains a need to better understand the role of religiosity as a potentially facilitating factor which may influence health seeking behaviors and how patient’s belief system may be more optimally used to enhance their recovery process.

### 4.2. Religiosity and 2-year mortality

After adjusting for several sociodemographic variables of prognostic importance, we found a higher risk of 2-year total mortality among those who reported praying for their health. However, this association was no longer statistically significant after accounting for patients’ clinical and psychosocial characteristics. There are several possible reasons why praying for one’s health might be associated with an increased risk of dying. Since prayers are a common form of complementary and alternative medicine [37], it is possible that in response to a life-threatening illness such as an ACS, patients with poorer health status who are at greater risk for worse health outcomes may engage in private religious practices, such as praying, as a coping mechanism during their recovery [38, 39]. Similarly, our findings suggest that the higher unadjusted risk of dying at 2 years after hospital discharge for an ACS among patients who prayed for their health may be attributable to the potential confounding effects of the severe symptoms of depression and anxiety, previously diagnosed comorbidities, and higher GRACE risk scores which were found to be more prevalent among those who prayed for their health compared with those who did not make petition prayers for their health. Another possibility is that if those who prayed for their health had a passive dimension of “spiritual health locus of control” believing that a higher power had complete control over their health, [40] they may have been less likely to engage in their healthcare or adopt recommended lifestyle changes. In support of this claim, in a prior publication using the TRACE-CORE data we observed that those who reported praying for their health had lower levels of patient activation (likelihood of engaging with their healthcare) compared with those who did not pray for their health [41]. Our findings underscore the need for future research to better understand how religious beliefs and practices may hinder patient engagement with their healthcare and impact survival outcomes.

We initially found a significantly higher risk of dying at 2 years following hospital discharge for an ACS among patients who acknowledged deriving strength and comfort from religion compared with those who reported none; however, this association was no longer statistically significant in the fully adjusted regression model. Our findings are in contrast with the results from an earlier study conducted in the 1990’s of 232 middle-aged and older US adults who underwent CABG surgery which observed that participants who did not derive strength and comfort from religion had three times the risk of dying from any cause during the subsequent 6 months compared with those who reported deriving at least some strength and comfort from religion [42]. In the Women’s Health Initiative Observational Study, 87% of 92,395 participants self-reported deriving strength and comfort from religion, however after accounting for various potentially confounding variables, there was no association between strength and comfort from religion and all-cause mortality [43]. Future in-depth qualitative interviews can be used to explore the role of strength and comfort from religion in healthy populations and among patients dealing with a life-threatening illness, to better understand how the receipt of strength and comfort from religion may influence their overall well-being and survival.

The use of intercessory prayers for healing and recovery from illness has been examined in several studies with inconclusive results of either no association, better, or worse health outcomes associated with the receipt of prayers made by others for their recovery [44, 45]. In a clinical trial of 748 patients who underwent a PCI and were randomized to receive intercessory prayers or not, no between group differences in either mortality or hospital readmissions after 6 months were observed [46]. Likewise, we did not observe an association between awareness of intercessory prayers being made for one’s health and 2-year all-cause mortality. While the scientific rationale of possible health benefits associated with distant intercessory prayer has been questioned, proximal intercessory prayers have a clearer scientific basis through mind-body mechanisms [45]. The measure of intercessory prayers in the present study did not consider the physical and socio-emotional proximity of those praying for patients, which may be an important consideration for future studies.

Several religious doctrines have been shown to facilitate the adoption of healthier behaviors and discourage actions such as cigarette smoking and excessive alcohol consumption with potential harm to the body [6, 7]. In the present study, most participants who reported religious engagement were more likely to be non-smokers and to report moderate to no use of alcohol. Despite these findings, we did not observe any protective effect of religiosity on overall survival. There remains an ongoing need for a more in-depth understanding on how religiosity may influence cardiovascular risk, survival, and post-discharge outcomes among patients discharged for an ACS.

### 4.3. Study strengths and limitations

Data used in the present investigation were obtained from a large prospective study of a socio-demographically diverse patient cohort, enhancing the generalizability of our findings. We analytically accounted for several sociodemographic, psychosocial, and pertinent clinical variables which could influence post-discharge survival. The extent of patients’ religious engagement was assessed by 3 separate items providing a unique opportunity for understanding the association between religiosity and survival following hospitalization for an ACS. Although we observed no significant association between our measures of religiosity and the multivariable adjusted risk of 2-year all-cause mortality, this study provides contemporary findings on the use of religious beliefs and practices for health among patients hospitalized for an ACS.

Our findings should be considered in light of several potential limitations. With respect to measurement error, the life-threatening experience of an ACS may have caused patients to over-report the extent of their religious beliefs and practices, which may have introduced non-differential misclassification of patient’s religiosity. There was no ascertainment of the duration of engagement in the reported religiosity measures, which is an important consideration since these are not static behaviors but may change in response to the patient’s physical and mental health status. We did not assess other coping strategies used by our study participants that may also influence their overall well-being and survival. In addition, we did not have information on patients’ religious affiliation and rituals/practices and could not explore whether the association between religiosity and mortality post-discharge for an ACS differed according to religious affiliation or rituals. Lastly, despite adjusting for several important potentially confounding variables of prognostic importance, there remains the possibility for residual and unmeasured confounding given our observational study design.

## 5. Conclusions

In this prospective cohort study, the majority of hospital survivors of an ACS derived strength and comfort from religion, prayed for their health, and were aware of others praying for their health. Most participants who reported religious involvement were more likely to engage in healthy lifestyle behaviors. Although religiosity did not influence long-term survival after an ACS, healthcare providers should recognize that patients use their religious beliefs and practices as strategies for improving their health status as this would contribute to a holistic approach to patient management and promote cultural competence in clinical care. Future longitudinal studies are needed to evaluate the religious beliefs and practices of patients and how this may influence disease self-management following hospitalization for an ACS, including evaluating whether patients recovering from an ACS may be experiencing religious struggle or negative coping, which may lead to adverse short and/or long-term health outcomes.
